# How can we reach the target of glucose control in type 1 diabetes?

**DOI:** 10.1111/1753-0407.13413

**Published:** 2023-05-22

**Authors:** Zachary Bloomgarden

**Affiliations:** ^1^ Department of Medicine, Division of Endocrinology, Diabetes and Bone Disease, Icahn School of Medicine at Mount Sinai New York New York USA

The convergence of improved continuous glucose monitoring (CGM) systems with effective automated insulin delivery (AID) controlled insulin pump therapy offers the promise to bring treatment of type 1 diabetes (T1D) to a formerly unavailable level of success.

CGM has become crucial in the management of people with T1D. A metaanalysis of 21 randomized controlled trials (RCT) of 2149 persons with T1D comparing CGM with capillary glucose monitoring showed significant 0.23% reduction in glycated hemoglobin (HbA1c),^1^ and a metaanalysis of 75 observational studies of 28 063 persons with T1D using CGM showed 0.4%–0.6% reduction in HbA1c, sustained up to 2 years; in both analyses the degree of reduction was proportional to the baseline HbA1c level.^2^ A recent analysis from the Diabetes Prospective Follow‐up initiative of 32 117 people with T1D, ranging in age from 1 to 25 years, from 511 diabetes centers in Austria, Germany, Luxembourg, and Switzerland, compared 10 883 using CGM with 21 234 only using capillary blood glucose monitoring.^3^ Severe hypoglycemia occurred at rates of 7 versus 9, and hypoglycemia‐induced coma at rates of 1 versus 2 per 100 person‐years respectively, and diabetic ketoacidosis (DKA) with pH <7.3 occurred at rates of 4 versus 7 and DKA with pH <7.1 at rates of 0.4 versus 0.9 per 100 person‐years, respectively, giving ~50% reductions in the rates of the most serious acute metabolic complications. Among persons using CGM, those with lower mean glucose and higher time below the target glucose range of at least 70 mg/dL had, as expected, more hypoglycemia, but another CGM parameter predicting hypoglycemia was the degree of glycemic variability as the percentage coefficient of variation (SD divided by the mean). DKA with pH <7.3 (but not as strongly that with pH <7.1) increased linearly with mean glucose and with time above the target glucose maximal level of 180 mg/dL, while showing no correlation with glycemic variability. The authors of this study concluded, “The effectiveness of continuous glucose monitoring to reduce life‐threatening acute diabetes complications provides evidence to advocate its use in patients with insulin therapy,” leading practitioners to realize that its use now is part of the standard of care for T1D. Indeed, the use of CGM increased, with analysis of ~25 000 participants in the T1D exchange showing that 6% used CGM in 2011, increasing progressively to 38% in 2018.^4^ Racial disparities remain, however, with analysis of approximately 100 Hispanic, 100 non‐Hispanic White, and 100 non‐Hispanic Black persons with T1D showing 58%, 53%, and 31% using CGM, respectively, after adjustment for multiple demographic factors.^5^

As another argument for widespread use of CGM, although HbA1c is an effective tool for the ascertainment of glycemic control in populations of persons with T1D, there are high levels of variability in the relationship between HbA1c and mean plasma glucose from individual to individual.^6^, ^7^ In an important fashion, the use of CGM allows the practitioner to have a greater recognition of the accuracy of HbA1c as a measure of the degree of glycemic control of a given patient, with the Glucose Management Indicator (GMI) calculation based on mean glucose^8^ clarifying whether a given individual exhibits a high, normal, or low degree of hemoglobin glycation for their glycemic level. Recognizing this, serial HbA1c values of a person with T1D may be seen to reflect that individual's glycemic stability.^9^ Measures of HbA1c variability based on the standard deviation of the HbA1c level in a given person (A1c‐SD), indicating fluctuations in that individual's degree of glycemic control over extended periods, appear to be of great importance as measures of risk of adverse T1D outcome.^10^ Examples of this are in analysis of participants in the Diabetes Control and Complications Trial, with the top quintile of A1c‐SD having a 2.5‐fold greater likelihood of retinopathy progression than those in the first quintile,^11^ with similar findings for albuminuria, retinopathy, and cardiac autonomic neuropathy among adolescents with T1D,^12^ and, recently, in a study of development of cardiovascular disease during 14‐year follow‐up of participants with T1D in the Coronary Artery Calcification in Type 1 Diabetes (CACTI) study.^13^

An increasingly important approach involves use of CGM to control insulin dosing via insulin pump with AID. The dosing algorithms required for such systems are complex. With subcutaneous glucose as the main “input,” the ideal AID system would take into account a variety of factors including “sensor noise” due to variability in readings, the time lag between blood glucose and the measured glucose and the additional time lag required for the CGM device to generate a glucose value, then the absorption kinetics of subcutaneous insulin, meal factors leading to variability in gut carbohydrate absorption, hepatic glucose production, and effects of physical activity and the effects of a variety of stress hormones and cytokines on insulin action.^14^ Recognizing that many of these factors are beyond the scope of current technology, existing AID systems have focused on control of basal (rather than meal‐related) insulin requirements. Three types of algorithms have been proposed.^15^ Proportional‐integral‐derivative control systems such as the one used in the Medtronic systems^16^ use three “signals,” one proportional to the current blood glucose level, one proportional to the integral of the blood glucose difference from predicted (the “error”) over time, and one proportional to the rate of change of the blood glucose. An approach termed model predictive control is used in the Insulet Omnipod system,^17^ with a mathematical model predicting future blood glucose states and insulin outputs, then calculating a control insulin input to minimize a cost function based on the difference between predicted and goal blood glucose levels, as well as the rate of change of the blood glucose and the insulin dose administered. An intriguing approach termed “fuzzy logic” represents current and predicted glucose levels and their first and second derivatives as ranges without sharp boundaries, conceptualized as low, normal, or high.^18^ Such a system has been illustrated in several in silico models,^19^, ^20^ and may offer promise for future development.

The evidence that AID systems can successfully improve glycemic control in people with T1D is quite encouraging. These systems have been demonstrated effective in several RCTs. A 13‐week RCT of 102 children ages 2–6 randomized to closed‐loop insulin‐delivery showed an increase in time in range of 70–180 mg/dL from 57% to 69%, with a 5% lower time above 250 mg/dL, an 18 mg/dL lower mean glucose and a 0.4% lower HbA1c.^21^ A 4‐week crossover RCT of 20 adults showed an increase in time in range from 58% to 62%, with HbA1c mean 7.5% with standalone pump and 7.1% with closed‐loop insulin delivery.^22^ In a meta‐analysis of 3 RCTs of 369 people with T1D ages 2–72, time in range increased from 57% to 70%, with particular improvement overnight, mean glucose decreasing to 173 versus 161 mg/dL from 6 am–11:59 pm, and 172 versus 147 mg/dL from midnight to 5:59 am, and with significant reduction in time <70 mg/dL from 2.2% to 1.8%; the greatest improvement occurred in those with lowest time in range.^23^ Observational studies have similarly shown benefit. In a 12‐month observational study of 18 older persons with T1D (mean age 74) starting a closed‐loop system, time in range increased from 64% to 80% and HbA1c decreased from 7.6% to 7%.^24^ For 46 adults with T1D transitioning to a closed‐loop system, HbA1c decreased over 6 months from 6.9% to 6.7%, with improvement in awareness of hypoglycemia, particularly in those with impaired perception of hypoglycemia symptoms.^25^ A registry of 46 043 people with T1D aged <1 to <26 years from 416 diabetes centers in Germany, Austria, Luxemburg, and Switzerland reported an increase in sensor‐augmented pump plus AID use from 32% in 2018 to 50% in 2021, with AID use increasing from 4% to 17%, and with improvement in time in range by 3% in those below age 6 years and by 5% in those age 6–26 years, in association with significant improvement in HbA1c in those age 11–26 years.^26^ For 1025 people with T1D from Argentina, Brazil, Chile, or Colombia who had transitioned from insulin pump with sensor to a closed‐loop system, mean sensor glucose was 144 mg/dL with coefficient of variation (the standard deviation divided by the mean) 34% and GMI 6.7%.^27^

The past 5 decades have witnessed dramatic improvement in the treatment of T1D. Studies from Finland show 30‐year mortality around 50% for those developing diabetes in 1964–1971, around 40% for diabetes developing in 1972–1979, and around 15% for diabetes developing in the 1980s, with limited data suggesting even greater improvement for those developing diabetes after 1990.^28^ The use of CGM and AID technologies promises to produce still greater improvement. Our challenge is to provide the best achievable level of care for all persons with T1D, in all socioeconomic and racial and ethnic groups, and in all countries.
